# On-chip generation of microbubbles in photoacoustic contrast agents for dual modal ultrasound/photoacoustic in vivo animal imaging

**DOI:** 10.1038/s41598-018-24713-4

**Published:** 2018-04-23

**Authors:** Dhiman Das, Kathyayini Sivasubramanian, Chun Yang, Manojit Pramanik

**Affiliations:** 1 School of Chemical and Biomedical Engineering, Nanyang Technological University, 62 Nanyang Drive, 637459 Singapore; 2 School of Mechanical and Aerospace Engineering, Nanyang Technological University, 50 Nanyang Avenue, 639798 Singapore

## Abstract

Dual-modal photoacoustic (PA) and ultrasound (US) contrast agents are becoming increasingly popular in recent years. Here, a flow-focusing junction based microfluidic device is used for the generation of nitrogen microbubbles (<7 μm) in two photoacoustic contrast agents: methylene blue (MB) and black ink (BI). The microbubble diameter and production rate could be precisely controlled in both MB and BI solutions. Microbubbles were collected from the outlet of the microfluidic device and optical microscope was used to study the size distributions in both solutions. Next, the microbubbles in both solutions were injected into tubes for phantom imaging experiments. Signal to noise ratio (SNR) of both US, PA imaging experiments were calculated to be 51 dB, 58 dB in MB + microbubbles and 56 dB, 61 dB in BI + microbubbles, respectively. Finally, the microbubbles were injected into the urinary bladder of rats for *in vivo* animal imaging. The SNR in US imaging with MB + microbubbles and BI + microbubbles were 41 dB and 48 dB, respectively. Similarly, the SNR in PA imaging with the same solutions were 32 dB and 36 dB, respectively. The effect of size and concentration of microbubbles in both MB and BI solutions, on the US and PA signals, has been examined.

## Introduction

Photoacoustic (PA) imaging has garnered a lot of attention from the biomedical researchers in recent years due to its high spatial resolution, greater depth of imaging, and wide imaging range from organs to organelles^1–4^. It has been used in a diverse range of applications such as imaging of neo-angiogenesis^5^, urology^6^, ophthalmology^7^, tumor^8^ imaging and many others^9–12^. Unlike ionizing X-ray imaging, this hybrid imaging modality poses no health hazard as it depends on the generation of acoustic waves on absorption of non-ionizing electromagnetic waves by a pulsed laser. Upon laser irradiation, the thermoelastic expansion occurs, leading to a small temperature rise which leads to the generation of the acoustic waves which can be detected with a broadband ultrasound transducer. PA signals are generated due to the absorption of light by endogenous chromophores like blood, melanin, lipids, and even water. When the contrast from the endogenous chromophores are insufficient, exogenous contrast agents are used, *e.g*., organic and inorganic dyes, nanoparticles etc.^13–18^. In the visible wavelength range, the penetration depth of light is less, limiting the imaging depth for certain clinical applications. To overcome this challenge, higher wavelengths [near infra-red (NIR) wavelength window] light are commonly used. In the NIR window the absorption and scattering effects are less in biological tissue leading to higher imaging depth, but with compromised contrast.

PA imaging has been integrated with other imaging modalities, such as ultrasound (US), magnetic resonance imaging (MRI), fluorescence etc. In particular, PA imaging systems can be easily integrated with US imaging systems, as they both rely on ultrasound transducers (UST) for data acquisition and image formation. Till now, most of the photoacoustic imaging systems used are either custom made research systems which doesn’t give access to raw channel data^19^ or clinically incompatible system which uses single element UST for data acquisition^20^. Recently, commercial clinical ultrasound imaging systems are available which allows dual modal US-PA imaging^21–24^. So, there is an increasing need for dual mode contrast agents in US-PA imaging. Dual-modal US/PA imaging systems have found applications in identifying tumors in lungs^25^. US imaging was used for *in vivo* identification and monitoring of orthotopic lung tumors whereas PA imaging was used to characterize oxygenation in the tissues surrounding the tumor. These agents have also been used for imaging sentinel lymph nodes^11,19^ in patients with breast cancer. US enables visualization of lymph node size and shape while PA helps to determine whether a lymph node is a sentinel lymph node by imaging the accumulated dye. Other examples include imaging of thyroid glands^26^, joints^27^ and angiogenesis^28^. Instead of dual-modal US/PA imaging, co-administration of both microbubbles and PA contrast agent dyes can be done but if the microbubbles are injected after the PA dye injection, the water phase of the microbubbles will eject the PA dye from the region of interest. Alternatively, we may wait for some time for injecting the microbubbles after the PA dye or vice versa. However, this may delay the patient’s diagnosis and there is no set time limit for such a procedure. Another substitute to co-administration is pre-mixing the microbubbles with the drug payload such as dyes, nanoparticles, etc. in the syringe itself just prior to injection. However, we cannot ensure how uniformly the drug payload will disperse in the water phase of the microbubbles. Moreover, the size distribution of the microbubbles may change from the original size distribution in the mixing process. Commercial ultrasound contrast agent such as SonoVue is sold in a sealed syringe by the manufacturer. For clinical purposes, the ingredients in the SonoVue solvent cannot be pre-mixed with FDA approved dyes such as methylene blue prior to injection. Thus, US/PA dual-modal contrasts, providing combinatorial diagnosis, using commercially available ultrasound platforms have become increasingly important^29–31^.

Microbubbles with a stabilized shell comprising of surfactants^32^ or phospholipids^33,34^ are used as US contrast agents^35^ for diagnostic applications, such as imaging the liver and heart^36^. These microbubbles may have different gas cores like nitrogen, perfluorobutane, etc.^37^. For US/PA based dual modal imaging, microbubbles have been integrated with several exogenous contrast agents. For instance, microbubbles have been shelled with porphyrin^38^ and gold nanoparticles^39^, synthesized in methylene blue^30^ solution as well as encapsulated with black ink^40^. Traditionally, microbubbles are produced using bulk mixing methods like mechanical agitation or sonication^41^. However, the bubbles produced through these methods are polydisperse. A narrow size distribution is desirable because the resonance frequency of the microbubbles depends on its diameter^42^.

We looked into the generation of microbubbles using microfluidics with a flow-focusing junction^34,43,44^. There are several advantages of using microfluidics for generating microbubbles. Firstly, the diameter, production rate, and the number of microbubbles to be delivered can be accurately controlled, which can be used to regulate how much microbubbles are to be released *in vivo* for targeted drug delivery^45^. Secondly, microfluidic devices can be miniaturized for placement within a catheter so that the microbubbles can be directly injected into the therapeutic region of interest^41,44^. Thirdly, the gas shell composition of the microbubbles as well as the external liquid phase composition can be precisely customized. Microfluidics-produced microbubbles have been used for sonothrombolysis^46^, mouse tail injection^47^, etc. Microfluidic devices have also been used for the generation of nanobubbles for high resolution ultrasound imaging in the mouse aorta. We envisage the use of microfluidic devices for the continuous generation of nanoparticle coated monodisperse-microbubbles^48,49^ as dual modal US/PA contrast agents. However, only a small number of nanoparticle-based therapeutics^50^ have been clinically approved so far due to its complexity and statutory regulatory requirements. Although nanoparticles or dye-based contrast agents can fully diffuse in the tissue, microbubbles are more suitable to endovascular imaging owing to its echogenicity and has its own advantages. When microbubbles in methylene blue solution or any dye-based solution is injected in the gastrointestinal tract, urinary bladder, etc., the microbubbles being lighter than the solution, will float up and will be closer to the wall. This can provide structural information of the wall which is important because cancer begins on the wall and consequently spreads from there.

In this work, we report the generation of nitrogen microbubbles using a flow-focusing junction-based microfluidic device in photoacoustic contrast agents, namely, methylene blue and black ink. Methylene blue^30^ is already a clinically approved dye. Black ink^51^ was taken to further demonstrate the ability of microfluidic devices to generate monodisperse microbubbles once the gas and liquid flow rates^44^ were optimized. Several works^34,52^ have reported the generation of monodisperse microbubbles using polyoxyethylene glycol 40 stearate (PEG 40), glycerol and propylene glycol in microfluidic devices with good stability. In our work, we generated microbubbles using an external water phase containing methylene blue or black ink, in addition to PEG 40, glycerol and propylene glycol^34^. Stability analysis of the microbubbles in both solutions was first done using optical microscope. Thereafter, more microbubbles were generated and withdrawn into glass syringes which were injected into low density polyethylene (LDPE) tubes for phantom imaging. Finally, *in vivo* animal imaging was performed by injecting the microbubbles into the urinary bladder of rats.

## Results

### Microbubble size distributions

**Figure 1:**
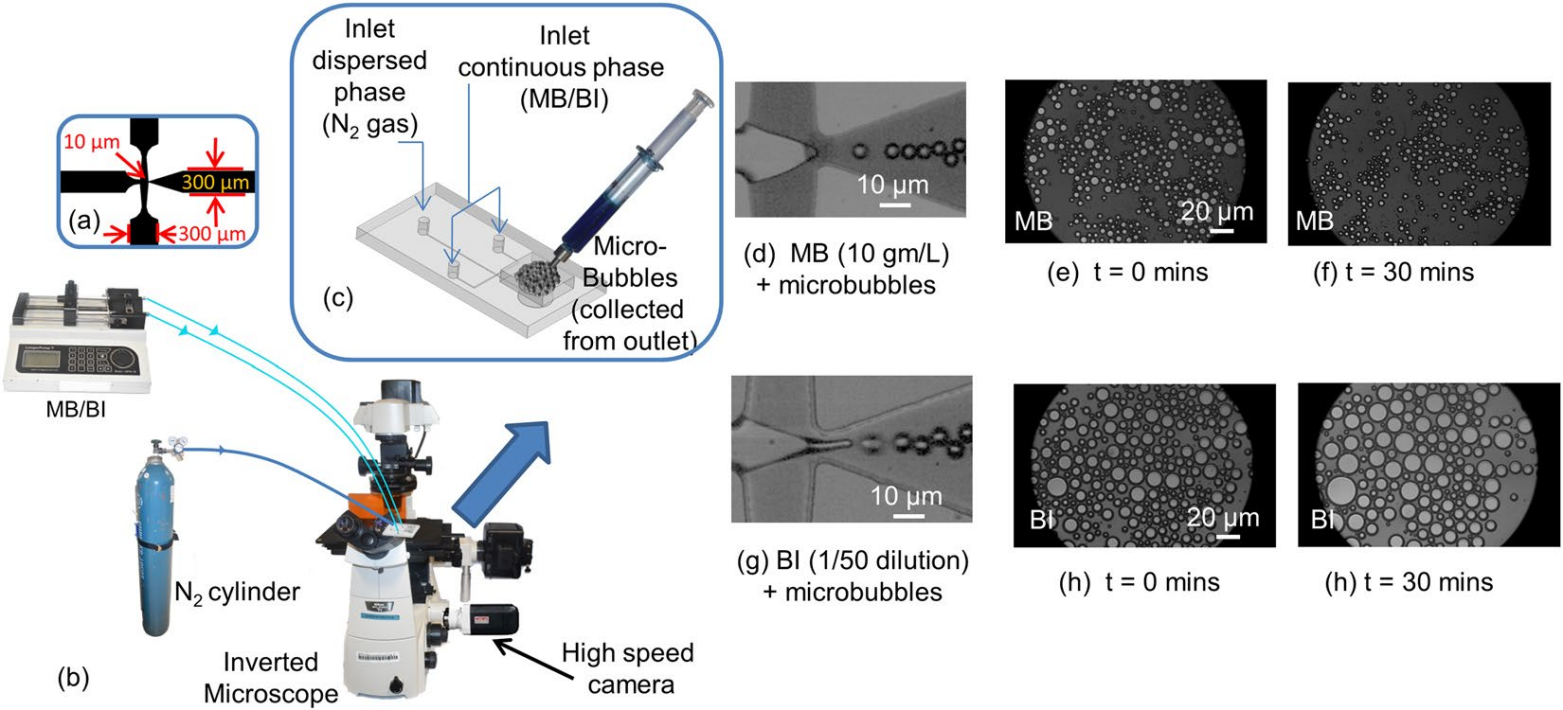
(**a**) Dimensions of the flow-focusing junction. (**b**) Schematic of the experimental set-up. (**c**) Microbubbles collected from the outlet of the microfluidic device. (**d**) and (**g**) show the monodisperse microbubbles generated in methylene blue (MB) and black ink (BI), respectively. (**e**), (**f**) shows the microbubbles in MB solution at time, t = 0 min and t = 30, min. (**h**), (**i**) shows the microbubbles in BI solution at time, t = 0 min and t = 30, min.

The top view of the microfluidic flow focusing junction, which was used for the microbubble generation, is shown in Fig. 1(a). A schematic of the experimental set up is shown in Fig. 1(b). This set up is described in detail in the Methods (1) section. At the flow focusing junction of the microfluidic device, microbubbles were generated in methylene blue (MB) [Fig. 1(a)] and black ink (BI) [Fig. 1(f)] solutions at flow rates of 2 ml/hr and 8 ml/hr, respectively. The difference in flow rates is due to the fact that with MB; 10% glycerol and 10% propylene glycol, was used compared to BI which contained 1% glycerol and 1% propylene glycol. A higher percentage of glycerol and propylene glycol is desirable as it retains monodispersity of the microbubbles^34^ but with BI, debris and pigments were observed at higher percentages at the flow-focusing junction of the microfluidic device under the microscope. As a result of this, the microbubbles production rate was not stable and it eventually led to choking of the microchannels.

At 25 °C, the viscosity of glycerol (947 centipoise) and polypropylene glycol (49.1 centipoise) is higher than the viscosity of water (1 centipoise). Hence, the higher percentage of glycerol and propylene glycol in the MB made it more viscous than the BI which are used as the continuous phases in our experiments. As a result, a lower flow rate had to be used for the MB compared to BI. Higher flow rates resulted in the leakage of MB from the microfluidic device. For BI, it was observed that at lower flow rates, the gas phase does not pinch off and hence, no microbubbles were formed. The production rates were in the order of 10^4^ microbubbles/sec and 10^6^ microbubbles/sec in the MB and BI, respectively. Also, the microbubbles population count was of the order of 10^8^ microbubbles/ml and 10^5^ microbubbles/ml for the MB and BI solutions, respectively. These were quantified by observing the flow-focusing junction using the high speed camera at 60,000 frames per second at 256 × 128 resolution as shown in Fig. 1(d,g). The counting was done just downstream of the flow-focusing junction. There is a slowdown of microbubble’s velocity downstream because the width of the microchannel expands to 300 microns as shown in Fig. 1(a).

**Figure 2:**
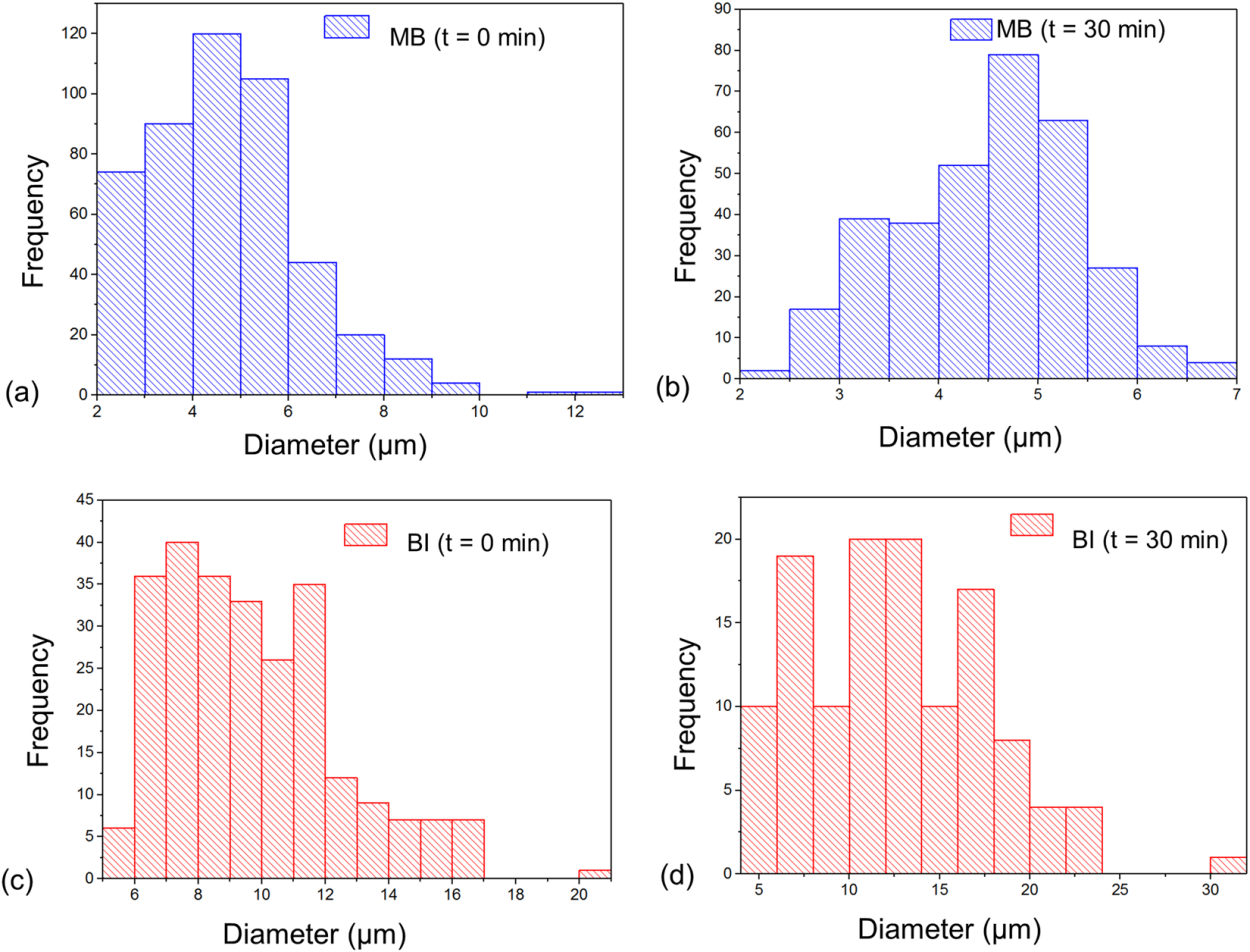
Size distributions of the microbubbles in methylene blue (MB) at (**a**) time, t = 0 min and (**b**) time, t = 30 min. Microbubbles in black ink (BI) at (**c**) time, t = 0 min and (**d**) time, t = 30 min.

The microbubbles were collected from the outlet as shown in Fig. 1(c) using glass syringes (Fortuna Optima, Singapore) and then pushed out between two cover slips for observation under the microscope. Initially, monodisperse nitrogen microbubbles of diameter 6 to 7 μm [Fig. 1(d,g)] were produced at the flow focusing junction for the both continuous phases. However, when the same batch of microbubbles was collected from the outlet and observed under the microscope, they were found to be polydisperse. Table 1 shows the mean, standard deviation, and polydispersity index (PI) of the microbubbles at time, t = 0 min and t = 30 min after the formation of the microbubbles. A plot of the size distribution of the microbubbles is shown in Fig. 2 at time, t = 0 min [Fig. 2(a,c)] and t = 30 min [Fig. 2(b,d)]. In the MB solution, 67% of the microbubbles had a diameter in the size range of 3 to 6 μm. After 30 minutes, it was observed that 70% were in the 3.5 to 5.5 μm range. Also, the PI of the microbubbles in MB solution is close to 1. So, the size of the microbubbles roughly remains the same. However, there is a 30% drop in the total number of microbubbles. As for the BI solution, 80% were in the size range of 6 to 12 μm. After 30 minutes, 85% were in the size range of 4.5 to 20 μm. Also, the PI of the microbubbles in BI solution is higher compared to the MB solution. The number of microbubbles reduced by around 50% as well. The polydispersity and loss in the total number of microbubbles occurred due to the coalescence and fragmentation of the same, which is regarded as Ostwald ripening effect. This effect is prevented by using a mixture of glycerol and propylene glycol. It has been reported that the monodispersity^53^ can be maintained by increasing the inter-bubble spacing. However, in our experiments, the microbubbles were withdrawn into glass syringes and pushed out between cover slips. So, the spacing between the microbubbles could not be controlled and consequently, the monodispersity was compromised.

**Table 1:** Mean, standard deviation (SD), and polydispersity index (PI) of the microbubbles in methylene blue (MB) and black ink (BI) and time, t = 0 min and t = 30 min.

	MB		BI	
	t = 0 min	t = 30 min	t = 0 min	t = 30 min
Mean diameter (μm)	4.72	4.50	9.73	12.58
SD	1.58	0.90	2.71	5.04
PI	1.11	1.04	1.08	1.16

### Phantom imaging

**Figure 3:**
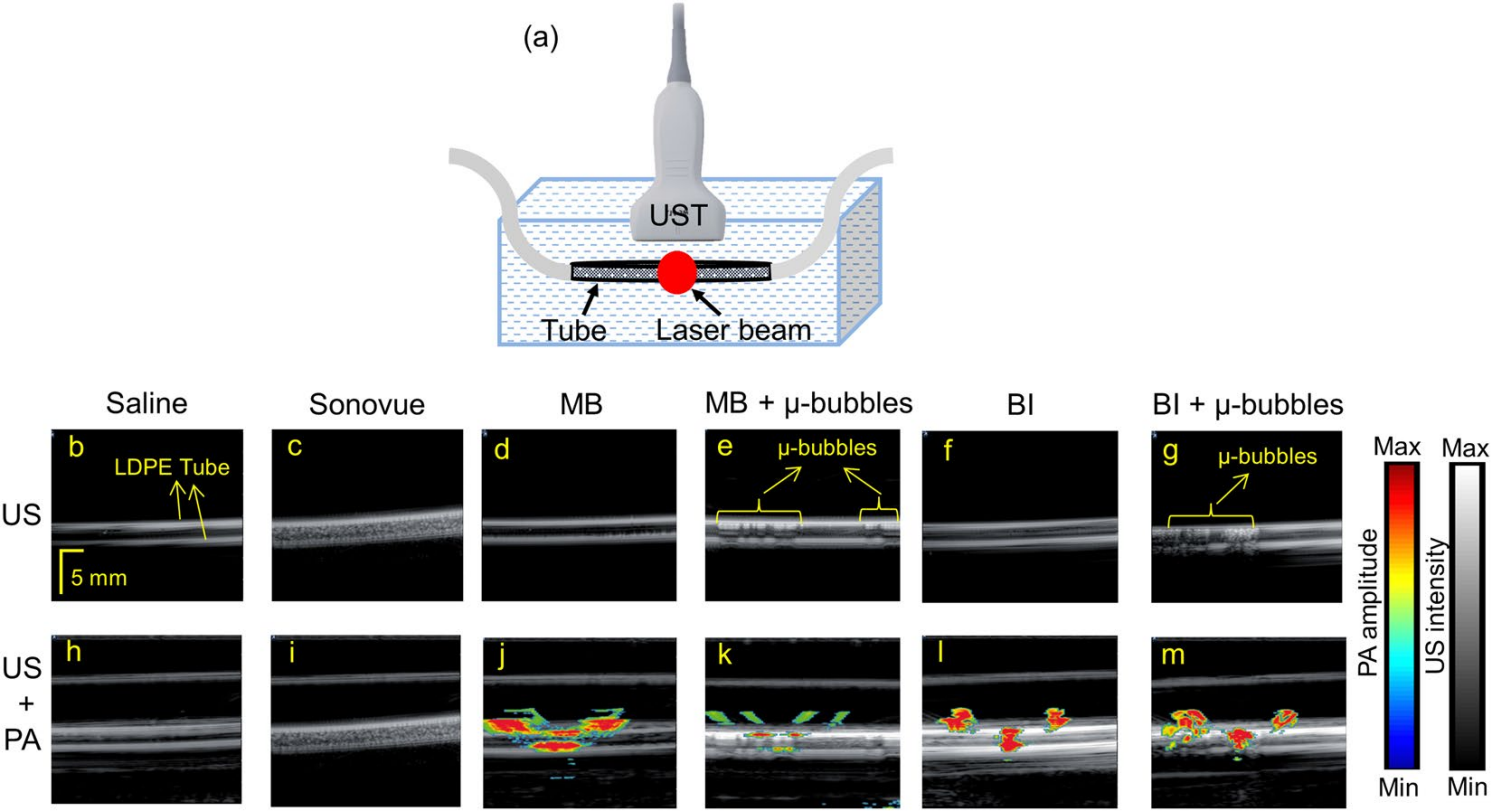
(**a**) Schematic representation of the low-density polyethylene (LDPE) tube with sample immersed in a water bath. The red circle on the tube denotes the area of laser illumination. (**b**–**g**) US images of saline, SonoVue, MB, MB + µ-bubbles, BI, BI + µ-bubbles, respectively, and (**h–m**) Combined US + PA images of saline, SonoVue, MB, MB + µ-bubbles, BI, BI + µ-bubbles, respectively. Scale bar and colour bar are added.

Microbubbles in MB and BI, were collected from the outlet of the microfluidic device and injected into LDPE tubes for phantom imaging. The US + PA signals obtained were also compared with different samples. Figure 3 shows the US and the combined US + PA images of the different samples obtained from the photoacoustic imaging system with clinical ultrasound platform. The detailed description of this set-up is given in Method (2). The different samples that were imaged includes saline as general control; SonoVue, a commercial ultrasound contrast agent as ultrasound control, plain MB and BI as control for PA imaging and finally, the test samples of MB + microbubbles and BI + microbubbles. Figure 3(b–g) show the US image of the tube containing saline, SonoVue, MB, MB + microbubbles, BI, and BI + microbubbles, respectively. Figure 3(h–m) show the combined US and PA images of the tube phantom containing saline, SonoVue, MB, MB + microbubbles, BI, and BI + microbubbles, respectively. The gray scale image represents the ultrasound image and the coloured region represents the PA image. In the LDPE tubes, the dual modal agents are not uniformly distributed. This is because when the agents are injected in to the tube, the exact flow and distribution of the microbubbles cannot be controlled precisely, which leads to the uneven distribution of the contrast agents. Additionally, we observed reflection artefacts of the edges of the tubes in the US signals and side-lobes in the PA signals. This is inherent to the clinical ultrasound system we used (E-cube). Removing these artefacts are challenging as we do not have access to the internal programming and settings of the program codes used by the manufacturer. Moreover, the PA signal is observed only in a small area because the laser illumination area (~1 cm) is small compared to the imaging area of the transducer (3.85 cm).

From saline (control) sample there is no noticeable US and PA signal as expected [Fig. 3(b,h)]. Only the ultrasound signal from the outline of the tube is visible as shown in Fig. 3(b). There is no US and PA signal from within the tube. From the commercial ultrasound contrast agent, SonoVue, very strong ultrasound signal can be noticed but there is no evident PA signal as shown from Fig. 3(c,i). MB, a well-established PA contrast agent gave no US signal but strong PA signal (laser excitation at 675 nm wavelength) as evident from Fig. 3(d,j). On the other hand, MB solution with microbubbles gave strong US and PA signal as seen in Fig. 3(e,k). BI sample gave no US signal but very strong PA signal at 1064 nm as shown in Fig. 3(f,i). However, one of the dual modal contrast agent BI with microbubbles gave very strong US and PA signal at 1064 nm as depicted in Fig. 3(g,m). It is to be noted that the ultrasound signal from the LDPE tube, as shown in Fig. 3(b), is visible in all the images and should not be confused with the US signal generated from the microbubbles. The US signals from the microbubbles are highlighted in Fig. 3(e,g). Additionally, it is to be noted that the bubbles have a tendency to float up and accumulate towards the top surface of the tube.

When the concentration of microbubbles is high, there is a very strong ultrasound signal from the microbubbles. Simultaneously, a weaker PA signal is observed from underneath the microbubbles in the tube. This is observed in Fig. 3(k,m). However, this effect is less pronounced in Fig. 3(m) because the total number of microbubbles was lesser in BI than in MB as stated earlier. The microbubble-based dual contrast agents have a synergistic effect as reported by Jeon *et al*.^30^, which mentioned that the presence of microbubbles reduced the PA signal. In our work, we observed that the SNR values do not change due to the presence of microbubbles in the MB and BI solutions. However, we clearly see a difference in the signal of the PA images area-wise between Fig. 3 (j) and (k) which was less pronounced between (l) and (m). Over time as the microbubbles dissolved or move away from the illuminated area, the area of the PA signal would also change.

The signal to noise ratio (SNR) for the various phantoms were calculated and shown in Table 2. SNR is given in decibels (dB) by *SNR* = 20 × *log*_10_(*V*/*n*), where $V$ represents the PA signal amplitude, $n$ stand for the background noise amplitude. The SNR is calculated after averaging 30 image frames. The saline solution had the least SNR values for both US and PA imaging, 0.7 dB and 2.5 dB, respectively. SonoVue had the maximum SNR for ultrasound signal (69 dB), but negligible SNR for PA imaging (3.5 dB). On the other hand, the SNR for US signal was 28% less for MB + microbubbles (51 dB) and 7% less for BI + microbubbles (61 dB) in comparison to SonoVue. The SNR for PA signal of the dual mode contrast agents was compared with the respective dyes and there was no significant change in the SNR for PA signal due to the presence of the microbubbles. The reduction in the PA SNR values was due to the presence of the microbubbles in MB (58 dB) and remained close to 60 dB in BI. There is no significant change in SNR as the microbubbles are not distributed uniformly throughout the tube. There are pockets in the tube in which the dyes are concentrated near the top surface of the tube. SNR for PA signal is calculated from the entire tube, which makes the SNR of PA signal from the MB + microbubble and BI + microbubble similar to that of pure MB and BI. However, if SNR is calculated from the PA signal right below the region where the bubbles are located there will be a noticeable difference.

**Table 2:** Comparison of signal to noise ratios (SNR) of US and PA signals for the various samples in tube phantom.

Sample	US (dB)	PA (dB)
Saline	0.7	2.5
SonoVue	69	3.5
Methylene blue (MB)	3.3	64
Methylene blue (MB) + microbubbles	51	58
Black ink (BI)	3.7	56
Black ink (BI) + microbubbles	64	61

### Urinary bladder imaging

**Figure 4:**
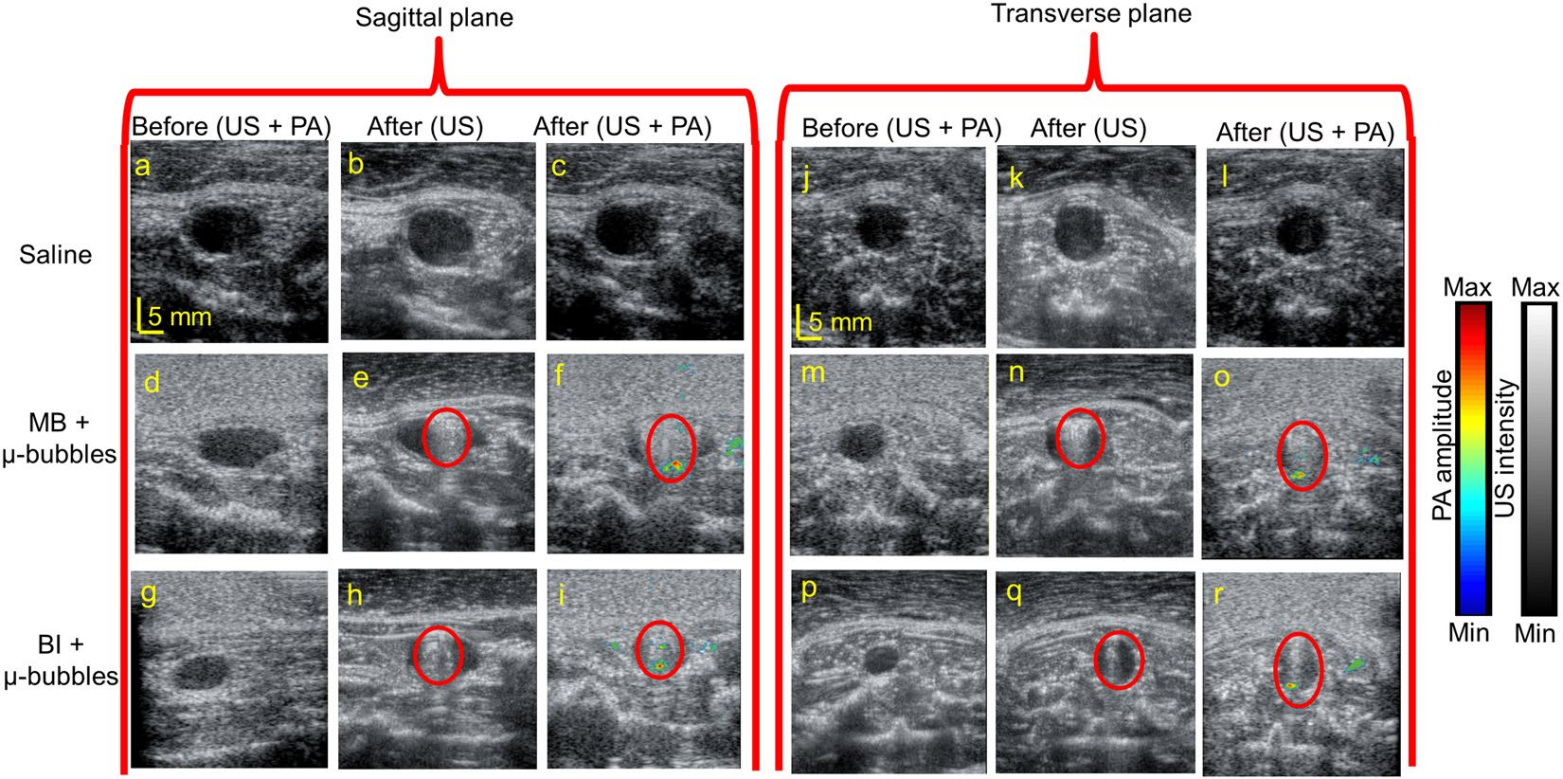
*In vivo* rat urinary bladder images (US + PA and US). Sagittal plane: (**a**–**c**) before and after injection of saline, (**d**–**f**) before and after injection of MB + µ-bubbles, (**g**–**i**) before and after injection of BI + µ-bubbles. Transverse plane: (**j–l**) before and after injection of saline, (**m**–**o**) before and after injection of MB + µ-bubbles and before and after injection of BI + µ-bubbles. Colour bar and scale bar are added.

For *in vivo* experiments, rat urinary bladder imaging was performed to demonstrate the use of dual mode contrast agents. The rat bladder was imaged in the transverse and the sagittal plane. Figure 4 shows the bladder imaging in both the planes before and after injection of saline, MB + microbubbles, BI + microbubbles. Before injection, combined (US + PA) images were obtained. After injection, US only and combined (US + PA) images were obtained. It can be noted from Fig. 4(a–c) and (j–l) that saline does not provide any US and PA signal although there is significant increase in the size of the bladder after injection images. Figure 4(d–f) and (m–o) show the before and after injection images of MB + microbubbles in sagittal and transverse planes, respectively. From the after injection images, it can be observed that there is a strong US signal from microbubbles and a strong PA signal from MB. The microbubbles float and move towards the top wall of the bladder. As a result, the US signal from the microbubbles is strong from the top part of the bladder. The PA contrast in the *in vivo* images appear as such because once injected into the urinary bladder, the microbubbles float at the top whereas the photoacoustic dyes will be settled at the bottom. The PA signal appears from the bottom part of the bladder wall as the dye stains the bladder walls. There are also stains of photoacoustic dyes visible along the ureter and urethra as can be seen in Fig. 4(f,i,o and r). Similar results were obtained with BI + microbubbles as observed from Fig. 4(g–i and p–r) showing the before and after injection images of the BI + microbubbles in sagittal and transverse planes. The SNR was calculated and shown in Table 3. For saline, the SNR of US and PA signal was observed to be negligible. On the other hand, the US and PA imaging SNR values for MB + microbubbles were 41 dB and 32 dB, respectively, and the SNR for US and PA imaging with BI + microbubbles were 48 dB and 36 dB, respectively. There was a significant difference between the SNR recorded from phantoms and *in vivo*. There was a difference between the SNR from animal experiments for ultrasound and PA for both the contrast agents in comparison to the phantom experiments. This occurs because there is lot of scattering and diffusion in the tissue.

**Table 3:** Values of signal to noise ratios (SNR) of US and PA signals from the *in vivo* animal images in both sagittal and transverse planes was obtained and then, average was taken.

Sample	US (dB)	PA (dB)
Saline	4	9
Methylene blue (MB) + microbubbles	41	32
Black ink (BI) + microbubbles	48	36

### Effect of microbubbles on US + PA signal

With increase in concentration (number of microbubbles/ml) and microbubble diameter (μm), the mean intensity of the ultrasound images increases. As it can be seen from Fig. 2, the total count of microbubbles in MB is much higher than that of BI. Meanwhile, Table 1 shows that the mean diameter of the microbubbles in MB solution is 4.5 μm whereas the same is 12.58 μm in BI solution after 30 minutes. So, the microbubbles are also smaller in diameter in MB compared to BI. Hence, the US imaging SNR values of the *in vivo* images of the urinary bladder stays relatively the same for both MB and BI solutions in the presence of microbubbles. The US images of the urinary bladder are shown in Fig. 4(e,h) and (n,q). However, for the tube phantom data as shown in Table 2; it was observed that US imaging SNR values of the BI + microbubbles is much higher than MB + microbubbles. This may be attributed to the complex wall interactions between LDPE tubes which has a hydrophobic surface and microbubbles. A comparative analysis of the effect of MB and BI on microbubbles within LDPE tubes would also be needed which is beyond the scope of this work. In the context of the PA signal, Jeon *et al*. reported that the PA signal decreases with the presence of microbubbles. This agreed well with the PA imaging SNR values obtained from our phantom imaging experiments. However, PA imaging SNR value did not drop significantly as can be seen from Table 2 when comparing MB and MB + microbubbles as well as BI and BI + microbubbles. This was observed as microbubbles coalesced to get bigger which occasionally burst while being pushed along the LDPE tubes. Moreover, the bubbles did not distribute evenly throughout the illuminated area of the tube.

## Discussion

The novelty of this work is that a flow-focusing junction is used for the continuous generation of microbubbles in optically absorbing dyes. Monodisperse nitrogen microbubbles of diameters 6 to 7 μm were generated in two photoacoustic contrast agents: MB and BI in the microfluidic device. The monodispersity as well as the microbubble concentration (number of microbubbles per ml) in photoacoustic contrast agents could be precisely maintained. It is a very simple yet effective way to inject monodisperse microbubbles once the device is miniaturized and placed in line with a catheter. Even without device miniaturization, these agents may be used for imaging the tumors present in the subcutaneous or intradermal regions. These agents may also be useful for obtaining the profile of tumors in the gastrointestinal tract, stomach, colon, urinary bladder, etc. At the flow-focus junction, the nitrogen microbubbles production rate has a population count of the order of 10^8^ microbubbles/ml and 10^5^ microbubbles/ml for the MB and BI solutions, respectively. SonoVue, a commercial US contrast agent, has 10^8^ microbubbles/ml^54^ which is comparable to the production rate of our microbubbles. In the stability analysis; the optical microscopy was done on the microbubbles which were first withdrawn into glass syringes and pushed out between two cover slips. The microbubbles were not monodispersed after syringe injection when observed via optical microscopy. Hence, further work needs to be done for cross-linking the methylene blue molecules with the surfactants which gets adsorbed at the gas-water interface of the microbubbles. The microbubbles in both MB and BI solutions were first injected into LDPE tubes for phantom imaging experiments using a dual-modal clinical US/PA imaging system. Finally, *in vivo* animal imaging of rat’s urinary bladder was performed. US signals were seen from the top of the urinary bladder as the microbubbles tend to float upwards. PA signals were seen from the bottom part of the urinary bladder. The photoacoustic signal in the *in vivo* study only originates from what appears to be a single point target at the bottom of the bladder versus both the top of bottom of the target in the tube phantom images. The difference in the PA signals appear as such due to internal algorithms of the E-Cube Alpinion system. This occurred despite applying the same Time Gain Compensation (TGC) setting for both *in vivo* and phantom experiments. Another reason is that in the phantom experiments, the solutions are more spread out in the LDPE tubes whereas in the urinary bladder experiments, the dyes would settle down to the bottom. So, the spacing between the microbubbles is more pronounced compared to the urinary bladder experiments. Both the dyes have complete dissolved in their respective solutions. This can be confirmed from the microfluidic experiments where we do not see any deposition of debris in the microchannels. The effect of microbubbles, on both US and PA signals, was also examined in this work.

## Methods

### Microbubbles generation in methylene blue and black ink

High purity nitrogen gas (Leeden National Oxygen Ltd., Singapore) was used as a dispersed phase for the generation of the microbubbles. The microbubbles were generated in two different photoacoustic contrast agents which were used as the continuous phases. The first continuous phase contained MB hydrate (Sigma, Singapore) at a concentration of 10 gm/L in deionized (DI) water. First, a DI water mixture is prepared which contains 5% PEG-40 (Sigma, Singapore), 10% glycerol (Sigma, Singapore), 10% propylene glycol (Sigma, Singapore) and 1% polyvinyl alcohol (PVA) (Sigma, Singapore). Thereafter, 10 gm of MB hydrate is added per litre of DI water. For the BI solution, first BI was diluted 50 times in DI water. Thereafter, 5% PEG-40, 1% glycerol and 1% propylene glycol were added to the BI and DI water mixture. With 10 percent of propylene glycol and glycerol concentration in BI solution, debris was observed in the microfluidic channels. Only when the 10 percent concentration was reduced to 1 percent, the debris were no longer visible. The BI was purchased from a stationary shop in Nanyang Technological University, Singapore. In the absence of PVA, the microbubbles in the MB solution adhered to the walls of the glass syringe which would coalesce to form bigger bubbles over time and were getting destroyed post-syringe injection. We observed adding PVA to the BI solution was not necessary as the microbubbles did not adhere to the walls of the glass syringe, were still in the microscale and gave a good US signal. Flow rates of 2 ml/hr and 8 ml/hr were provided for the MB and BI solutions, respectively by a syringe pump (Longer) to the two inlets of the polydimethylsiloxane (PDMS)-based microfluidic device at the flow-focusing junction. Although the standard commercial vial of SonoVue contains 5 ml of solvents, the required dose varies depending on the part of the body which is to be examined. For instance, for the B-mode imaging of cardiac chambers in adults, the recommended dose is 2 ml. So, to produce microbubbles in 2 ml quantity of MB and BI, it will take 1 hr and 0.25 hrs, respectively. The PDMS-based microfluidic device was fabricated using standard soft-photolithography. The width of the flow-focusing junction was 10 μm and widths of all the inlets and outlets were 300 μm. The top view schematic of the microchannels is shown in Fig. 1(a). The height of the microchannels was 25 μm. For both MB and BI solutions, a pressure of 1 bar was delivered to the gas inlet of the microfluidic device from a nitrogen cylinder by using a pressure regulator (Spectron, Singapore). An inverted microscope (Nikon Eclipse TE2000) was used for observing the microbubbles generation at the flow focusing junction of the microfluidic device. The inverted microscope is fitted with a high-speed CMOS camera (Phantom Miro 310) which could record images at frames up to 650,000 frames per second (fps).

### Dual modal US-PA imaging system

**Figure 5:**
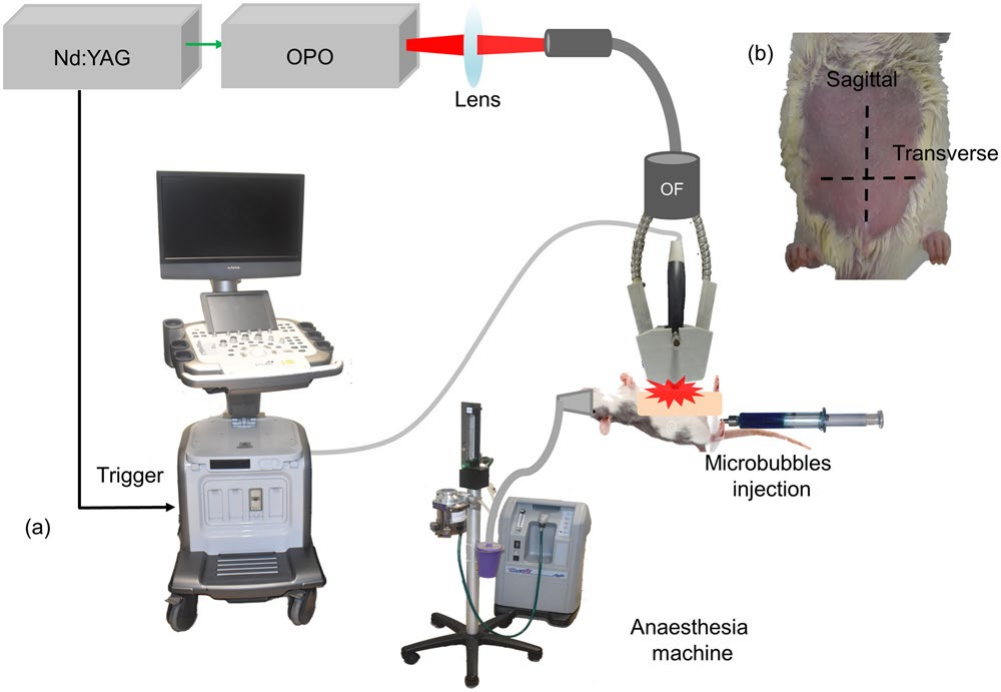
(**a**) Schematic representation of the photoacoustic imaging system with clinical ultrasound platform. OF, optical fiber bundle; USM, clinical ultrasound machine; UST, ultrasound transducer. The optical fiber holder houses the 2 output optical fiber bundle for illumination of tissue and ultrasound transducer for signal acquisition. An anaesthesia machine supplying isoflurane and oxygen was used to maintain the animal under anaesthesia for the duration of the experiments (**b**) Photograph of rat showing, sagittal and transverse imaging planes.

For combined dual modal US-PA imaging, a clinical ultrasound imaging system (E-CUBE 12 R, Alpinion, South Korea) was used. The system can be operated in US only mode, PA only mode, and combined US and PA imaging mode. A 128 element linear array transducer compatible with the clinical ultrasound imaging system is used for image acquisition. For US only mode the linear array transducer was used alone. The speed of sound is set at 1540 m/s and the centre frequency of the transducer is 8 MHz and the fractional bandwidth of 95%. The system has 64 parallel data acquisition hardware and combines the images from all the 128 channels to form a single image. The different parameters like depth of imaging, time gain compensation, frequency of transducer etc. can be set and modified on the user friendly control panel available on the ultrasound imaging system. For combined US and PA imaging, along with the ultrasound transducer, a light source for tissue excitation is used. A 10 Hz OPO laser (Continuum, Surelite OPO) was used as excitation source pumped by frequency doubled nanosecond pulsed Nd:YAG pump laser (Continuum, Surelite Ex, San Jose, California, USA) was used. The laser generates 5 ns pulses which can be tuned from 670 nm to 2500 nm. For PA imaging a trigger from the laser to the ultrasound machine is needed for data acquisition. This can be provided directly from the laser or using a photodiode. For each laser pulse fired data from 64 channels are acquired due to limitation in parallel data acquisition hardware. Therefore, for every two laser pulse, one image is obtained. Therefore, for PA imaging the effective frame rate is 5 frames per second (fps). The schematic of the imaging set up is shown in Fig. 5(a).

### Phantom imaging

To test the efficiency of the microbubbles in methylene blue and black ink as combined US and PA contrast agents, phantom imaging was done. For this, laser was tuned to 675 nm for microbubbles in MB solution, as MB has maximum absorption around this wavelength, and the laser was tuned to 1064 nm for imaging microbubbles in BI solution for demonstrating deep tissue imaging although BI has high absorption at most wavelengths in the visible and near infrared regions. Imaging at 1064 nm will be beneficial for deep tissue imaging as the penetration depth of light is high at this wavelength. Both the solutions containing microbubbles were injected in to LDPE tube of 1.68 mm inner diameter and the tube was placed in a water tank. Light illumination was done in free space and the area of illumination was approximately 1 cm. The transducer was placed parallel to the tube and perpendicular to the light illumination. US image was captured first, and then combined US and PA image was captured. The setup is shown in Fig. 3(a). For control saline, commercial ultrasound contrast agent, methylene blue solution and black ink solution was used. US only and combined US-PA images were captured for the same. The system allows to capture the images in 4 different data types namely radio frequency (RF), beam formed (BF), IQ demodulated format and scan converted (SC). For all phantom experiments, images were saved as beam formed data type and the imaging depth was set to 2 cm. 50 image frames were saved for each of the sample.

### Animal preparation and imaging

To prove the efficiency of the microbubbles in methylene blue and black ink as dual modal contrast agents *in vivo* imaging of rat urinary bladder was carried out. Animal experiments were performed in accordance with the approved guidelines and regulations, and were approved by the Institutional Animal Care and Use committee of Nanyang Technological University, Singapore (Animal Protocol Number ARF-SBS/NIE-A0263). Healthy adult female Sprague Dawley rats of weight 300 ± 50 g (aged 12–14 weeks) were obtained from InVivos Pte. Ltd., Singapore. To prepare the rats for imaging, firstly they were anesthetized with a mixture of Ketamine (85 mg/kg) and Xylazine (15 mg/kg). An intraperitoneal injection of 0.2 mL of the mixture is administered per 100 g of the rat body weight. Then, the abdominal area is cleared of hair using commercial hair removal cream. Subsequently, a 23 G urinary bladder catheter was inserted through the urethra and was secured using tissue glue to avoid leakage. Finally, to maintain the animal under anaesthesia during experiments 0.75% of isoflurane gas (Medical Plus Pte Ltd, Singapore); was given through a nose cone covering the nose and the mouth of the rat. Also, to monitor the heart rate and peripheral oxygen saturation of the rat throughout the experiments a pulse oximeter (Medtronic, PM10N with veterinary sensor, Minneapolis, Minnesota, USA) was attached to the hind leg of the rat. After experiments, the rats were euthanized with a pentobarbital overdose.

For animal PA imaging a handheld probe holder was used. To couple the laser light and the ultrasound transducer a bifurcated optical fiber was used (Ceramoptec GmbH, Germany). It has 1600 fibers in total with a numerical aperture of 0.22 and a diameter of 200 µm was used. The fiber and the transducer are fixed into a custom designed probe holder with three slots (centre slot for transducer and the two sides for optical fiber) forming the handheld probe for combined US and PA imaging. The animal is placed facing upwards and a 0.5 cm thick chicken tissue slice is placed on the shaved region to mimic the distance of the urinary bladder from the skin for humans. The probe is placed on the animal and both US and combined US + PA images are obtained before injection in the transverse and sagittal planes for control images [shown in Fig. 5(b)]. 0.8 mL volume of microbubbles in MB and BI solutions are injected through the catheter. US, and combined US + PA images of the bladder were obtained in the transverse and sagittal planes. Saline was used as control. All the images were saved as beam-formed datatype. Total imaging depth was set as 3 cm. 50 imaging frames were saved for each experiment.

### Laser safety

The optical fiber bundle has a coupling efficiency of ~50% at both 675 nm and 1064 nm laser wavelengths. At 675 nm the energy of light falling on the surface is 20 mJ per pulse with an illumination area of ~3 cm^2^ and 1064 nm the energy is 45 mJ per pulse with an illumination area of ~5 cm^2^. According to the American National Standards Institute (ANSI) the maximum permissible energy (MPE) on skin at 675 nm and 1064 nm are 20 mJ/cm^2^ and 100 mJ/cm^2^, respectively^55^. The fluence calculated for the experiments are 6 mJ/cm^2^ and 9 mJ/cm^2^ at 675 nm and 1064 nm, respectively. Therefore, we are well within the safety limit.
